# Analysis of Metabolite Differences in Different Tea Liquors Based on Broadly Targeted Metabolomics

**DOI:** 10.3390/foods13172800

**Published:** 2024-09-03

**Authors:** Xiongyu Li, Miao Niu, Hongyan Yang, Xianxiu Zhou, Jianliang Ding, Yawen Xu, Caiyou Lv, Jiahua Li

**Affiliations:** 1 College of Tea Science, Yunnan Agricultural University, Kunming 650201, China; lxy19912992159@163.com (X.L.); 2022210231@stu.ynau.edu.cn (M.N.); 13529725893@163.com (H.Y.); 2023210231@stu.ynau.edu.cn (X.Z.); 13937016027@163.com (J.D.); 15108721378@163.com (C.L.); 2 College of Pu-Erh Tea, West Yunnan University of Applied Sciences, Puer 665000, China; 1575859889@163.com

**Keywords:** Pu-erh tea, tea liquor, metabolomics, non-volatile compound

## Abstract

To expand the development of characteristic extension products of Yunnan tea and improve the utilization rate of Yunnan tea resources, in this study, we compared the metabolite composition among raw Pu-erh tea, ripe Pu-erh tea prepared with glutinous rice (according to tea to glutinous rice ratio of 1:3), and ripe Pu-erh tea prepared with a mixture of sorghum, rice, glutinous rice, wheat, and corn as raw materials (according to a tea to glutinous rice ratio of 1:3). Rice flavor liquor prepared with 100% glutinous rice served as a control. The raw Pu-erh tea liquor (RAWJ), ripe Pu-erh tea liquor (RIPEJ), ripe Pu-erh tea mixed grain liquor (HHLSJ), and rice-flavor liquor (MJ) were all brewed by semi-solid fermentation. The non-volatile components of the liquor samples were analyzed by ultra-high-performance liquid chromatography-tandem mass spectrometry as a broadly targeted metabolomics technique. A total of 691 metabolites were identified from the four samples. Among them, 674, 671, 633, and 667 species were detected in RAWJ, RIPEJ, HHLSJ, and MJ samples, respectively. Venn diagram analysis demonstrated 19, 21, and 14 unique metabolites in RAWJ, RIPEJ, and HHLSJ, respectively, compared with the metabolite composition of MJ. Flavonoids are the most important differential metabolite between tea liquor and rice-flavor liquor. This study provides a theoretical basis for the development of tea liquor products and offers insight into the difference in non-volatile components between tea liquor and rice-flavor liquor.

## 1. Introduction

Tea is one of the most popular and economically non-alcoholic beverages in the world, which can be divided into six categories according to the degree of fermentation: green tea (unfermented), white tea (slightly fermented), yellow tea (slightly fermented), oolong tea (semi-fermented), black tea (fully fermented), and dark tea (post-fermented) [1]. As a unique distilled spirit in China, Chinese liquor, also known as baijiu, exhibits a diverse range of flavors, which are categorized into 12 distinct types such as sauce-flavor, strong-flavor, mild-flavor, and rice-flavor liquors. These variations are influenced by diverse factors, including the specific regions where the raw materials are sourced, which contribute distinct characteristics to the liquor, as well as the fermentation processes [2]. Both tea and liquor have a deep historical heritage and remain highly desired consumer items in China and internationally.

Tea liquor (characterized by an alcohol content greater than 40%) is an extended product of tea that is brewed with grain water and tea as the main raw materials, which combines the advantages of tea and liquor, including the beneficial health effects of tea and the desired quality and flavor characteristics of liquor [3,4,5]. The brewing process of tea liquor is generally divided into liquid fermentation [6], solid fermentation [7], and semi-solid fermentation [8,9]. In recent years, there has been increased research interest in improving the fermentation technology of tea liquor along with attempts to uncover the functional properties of the tea liquor. Qin et al. [10] optimized the production technology of tea liquor fermentation by inoculating bacteria in black tea and rice wine; Xu et al. [11] developed a new type of fermented tea wine that is rich in anthocyanins using Ziyan tea as the main raw material. Wang et al. [12] optimized the production process of glutinous rice tea wine (alcohol content below 15%) based on a response surface method. Moreover, Li et al. [13] and He et al. [14] showed that tea wine is rich in catechins, gallic acid, and other active antioxidant substances, resulting in various health effects such as lowering blood lipids, preventing cardiovascular and cerebrovascular diseases, and improving immunity, suggesting that moderate drinking of tea wine could be beneficial to health.

Yunnan is the only producing area of Pu-erh tea, which can be divided into raw and ripe Pu-erh tea. Raw Pu-erh tea is prepared using the fresh leaves of the Yunnan large-leafed species of tea as the raw material, followed by fixation, rolling, and sun-drying, whereas ripe Pu-erh tea is prepared from the sun-dried green tea and is then processed according to a specific fermentation process [15]. According to the statistics of the Department of Agriculture and Rural Affairs of Yunnan Province and the China Tea Distribution Association, in 2023, the tea-planting area of Yunnan Province was 5.36 billion m^2^ and the total output of processed tea was 418,000 tons, representing an increase of 5.1% compared with the production levels of the previous year. However, the average price of Pu-erh tea is 168.3 CNY/kg, which is notably lower than that of other teas such as Longjing tea at 983.26 CNY/kg, resulting in a lack of motivation for tea farmers to invest in Pu-erh tea; consequently, it has become increasingly common for farmers to abandon the production of summer and autumn tea, seriously restricting the development of the Yunnan tea industry. The development of tea liquor and other processed tea products such as black tea bread [16] and Pu-erh tea paste [17] offers a conducive strategy to improve the utilization rate of Yunnan’s tea resources, increasing the scale and efficiency of the tea industry, and ultimately promoting the transformation and advancement of Yunnan’s tea industry by broadening consumption channels. In recent years, plant bioactive phenolic compounds have garnered widespread attention due to their myriad health benefits [18], and tea is abundant in phenolic compounds. Farag et al. [19]. utilized UPLC-MS and GC-MS for the analysis of metabolite fingerprints from four types of cinnamon.

To encourage such development, in this study, we used Pu-erh tea, glutinous rice, sorghum, rice, wheat, and corn as the main raw materials to prepare raw, ripe, and mixed-grain Pu-erh tea liquor, along with rice-flavor liquor, using semi-solid fermentation technology. The main metabolites of these samples were systematically analyzed and compared using ultra-high-performance liquid chromatography-tandem mass spectrometry (UPLC-MS/MS) combined with multivariate statistical analysis to comprehensively determine the main metabolic changes among the samples. The results of this study can therefore provide some theoretical support for the in-depth development of Yunnan Pu-erh tea liquor and other tea liquor products.

## 2. Materials and Methods

### 2.1. Raw Materials

Raw and ripe Pu-erh tea was obtained from Yunnan Yicang Trading Co., Ltd (Kunming, China). Sorghum was purchased from the Luancheng district Hebei Province Amoy grain shop (Luancheng, China). Rice and corn were obtained from Qiqihar North Kitchen Trading Co., Ltd. (Qiqihar, China). Glutinous rice was purchased from Wuchang Xingwang Rice Industry Co., Ltd. (Harbin, China). Wheat was obtained from Jincheng Pingle Flour Co. and Ltd. (Jincheng, China). Koji (small koji) was obtained from Angel Yeast Co., Ltd. (Yichang, China).

A refractometer was purchased from Hengshui Jinjun Trading Co., Ltd. (Hengshui, China). Alcohol meter Hengshui Green Trading Co., Ltd. (Hengshui, China). Electronic balance ME204/02 METTLER-TOLEDO Instrument Co., Ltd. (Zurich, Switzerland). Pure water (making tea liquor) was purchased from Kunming Zhen Ming Food Co., Ltd. (Kunming, China).

### 2.2. Preparation of Tea Liquor

The preparation process of tea liquor was based on the methodology described by Zou et al. [20] and the semi-solid fermentation method of the rice-flavor liquor followed the standard GB/T 10781.3-2006 [21]. We used the following process to slightly improve the production of raw Pu-erh tea liquor (RAWJ) and ripe Pu-erh tea liquor (RIPEJ). In brief, Angel liquor koji (0.6%) was added to cooked and cooled glutinous rice (1 kg); the mixture was then subjected to saccharification (30 °C, for 3 days). The tea soup (1.5 L) and tea leaves (330 g) were added to the glutinous rice-liquor mixture for secondary fermentation (28 °C for 7 days), followed by distillation (79–95 °C for 20 min at 20–25 mL/min) for liquor reduction. The ripe Pu-erh tea mixed-grain liquor (HHLSJ) was prepared with the same production process except for the use of 1 kg mixed grains (sorghum, rice, glutinous rice, wheat, and corn) rather than glutinous rice alone. The rice-flavor liquor sample, used as a control, was prepared in the same manner using glutinous rice as the raw material, although secondary fermentation was performed with pure water (Kunming Zhen Ming Food Co., Ltd.).

Fermentation was carried out in a stainless-steel distiller and fermentation barrel from Nanfeng County Daliang Brewing Equipment Factory using a constant-temperature fermentation box (LY small second layer; Dongguan Fenggang Lai Electrical Appliance Company, Dongguan, China). The fermentation conditions of all tea liquors were as follows: 0.6% koji, 1:3 tea-to-rice ratio, and 1:50 tea-to-water ratio of the secondary fermented tea soup (according to the national standard GB/T 23776-2018 [22] “Tea Sensory Evaluation Method”). The tea soup added amount was 1.5 times the mass of glutinous rice. The grain in the mixed-grain liquor was a crushed powder of 36% sorghum, 22% rice, 18% glutinous rice, 16% wheat, and 8% corn according to the standard GB/T 22211-2008 [23] of Wuliangye liquor, using Pu-erh ripe tea. The distillation method was based on the segmental liquor-receiving approach, with the first liquor receiving 60% alcohol by volume (ABV), and the last liquor having a volume below 45% alcohol by volume (ABV). The above processes were repeated three times and the alcohol content was standardized to 50% vol, with the volume of each bottle maintained at 500 mL. The samples were stored at −80 °C until analysis.

### 2.3. Sample Extraction

The metabolomics method was used to analyze the metabolite components of different tea liquors based on UPLC-MS/MS. The frozen and stored sample was thawed, 9 mL of this distillate sample was placed in a 50 mL centrifuge tube labeled with a unique code, and 300 μL of 70% methanol (chromatographically pure, Merck, Darmstadt, Germany) containing internal standard extraction solution was added to the tube facilitate dissolution. The mixture was vortexed for 15 min, followed by ultrasound treatment in an ice-water bath (0 °C, KQ5200E) for 10 min (ensure that the compounds in the sample are fully solubilized or released). The sample was then centrifuged at 12,000 rpm at 4 °C (5424R, Eppendorf, Hamburg, Germany) for 3 min, filtered through a microporous membrane with a pore size of 0.22 μm, and transferred to a sample bottle for storage until subsequent detection.

### 2.4. UPLC-MS/MS

The ExionLC™ AD (AB SCIEX Analytical Instrument Trading Co., Ltd., Shanghai, China.) and QTRAP 6500 MS/MS (Applied Biosystems, AB SCIEX, Shanghai, China) systems were used for UPLC-electrospray ionization (ESI)-MS/MS.

UPLC was performed with an Agilent SB-C18 column (1.8 µm, 2.1 mm × 100 mm). Mobile phase A was ultra-pure water (with 0.1% formic acid chromatographically pure, Aladdin Reagents Ltd., Shanghai, China) and mobile phase B was acetonitrile (with 0.1% formic acid added). The sample was measured using the following gradient procedure: 5%B at 0 min, which was increased linearly to 95% within 9 min, maintained at 95% for 1 min, decreased to 5% at 10–11.10 min, and then balanced at 5% to 14 min. The flow rate was 0.35 mL/min, the column temperature was 40 °C, and the sample volume size was 2 μL.

The operating parameters of the ESI source were as follows: source temperature of 550 °C, ion spray voltage of 5500 V (positive-ion mode) or −4500 V (negative-ion mode) and ion source gas I (GSI), gas II (GSII), and gas curtain gas (CUR) set to 50, 60, and 25 psi, respectively. The collision-induced ionization parameter was set to “high”. The QQQ scan was run in multiple reaction monitoring (MRM) modes with the collision gas (nitrogen) set to “medium”. The declustering potential (DP) and collision energy (CE) of each MRM ion pair were further optimized. A specific set of MRM (Figure S1) ion pairs was monitored at each period based on the eluted metabolites during each period according to the 3000 MRM product standard.

### 2.5. Qualitative Analysis of Metabolites

Qualitative analysis of metabolites was carried out according to secondary spectrum information (Figure S2), excluding isotopic signals, repetitive signals containing K^+^, Na^+^, NH_4_^+^ ions, and repetitive signals from fragment ions of other larger molecular weight substances. Detection of the relative content of species in different samples was performed using five parameters of substance detection (DP, CE, retention time, Q1, and Q3) to obtain qualitative data on the detected substances [24]. There are 3 levels for metabolite characterization: Level: Substance identification level, 1: sample substance secondary mass spectra (all fragmentation sub-ions of the substance), RT, and database substance matching score of 0.7 or more; 2: sample substance secondary mass spectra (all fragmentation sub-ions of the substance), RT, and database substance matching score of 0.5–0.7; 3: sample substance Q1, Q3, RT, DP, and CE checking consistency with the database substances.

### 2.6. Statistical Analysis

Analyst 1.6.3 software was used to process the mass spectrum data. Microsoft Office Excel 2019 was used for data processing with an online data analysis platform (https://cloud.metware.cn/). The statistical function in R (www.r-project.org) was used for unsupervised principal component analysis (PCA). The cor function of the R software package (version 3.5.1) was used to calculate Pearson correlation coefficients and determine their significance, with the data visualized in heat map form. The differences in metabolites between the two groups were analyzed using the projected variable importance (VIP) score according to a threshold of VIP ≥ 1 (|log2 fold change (FC)|) ≥ 2.0 or <0.05 to indicate a significant difference. VIP values (including score plots) were extracted from orthogonal partial least squares discriminant analysis (OPLS-DA) and permutation plots were generated using the R software package MetaboAnalystR (ggplot2 3.3.0).

## 3. Results and Discussion

### 3.1. Overview of Non-Volatile Metabolites in the Four Samples

**Figure 1:**
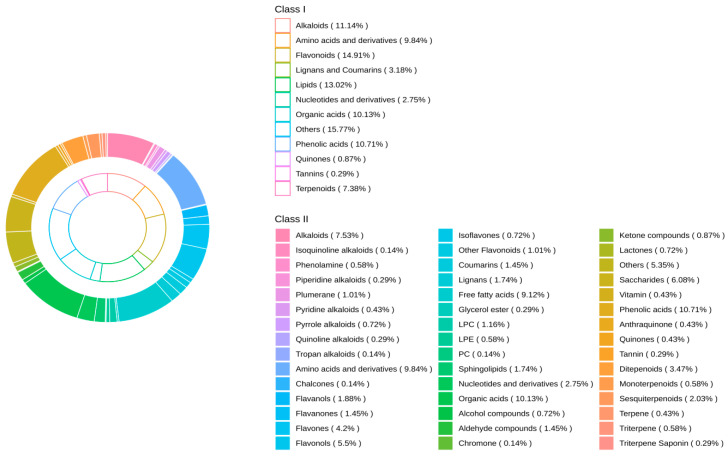
Ring chart of metabolite component proportions in the four liquor samples.

The metabolomics method was used to analyze the metabolite components of different tea liquor samples. Based on the UPLC-MS/MS detection platform, a total of 691 metabolites were identified in the RAWJ, RIPEJ, HHLSJ, and MJ samples. The proportions of each component in the four sample liquor are shown in Figure 1, including others (15.77%), flavonoids (14.91%), lipids (13.02%), alkaloids (11.14%), phenolic acids (10.71%), organic acids (10.13%), amino acids and derivatives (9.84%), terpenoids (7.38%), lignans and coumarins (3.18%), nucleotides and derivatives (2.75%), quinones (0.87%), and tannins (0.29%).

**Figure 2:**
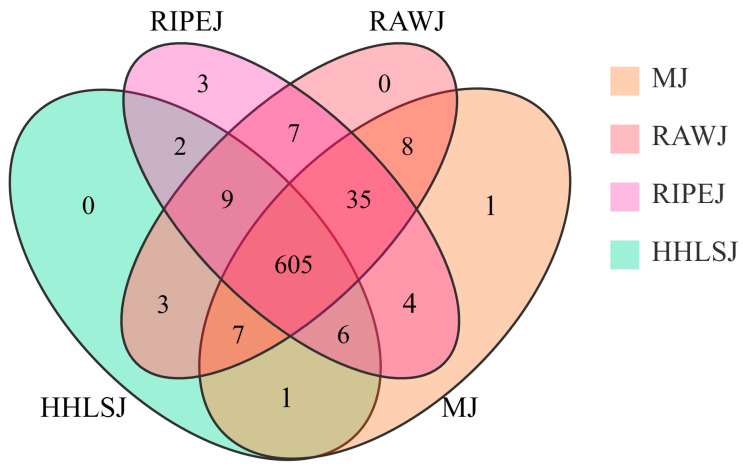
Venn diagram of the metabolites identified in the four liquor samples.

To clarify the differences between metabolites of the tea liquor and rice-flavor liquor metabolites, a Venn diagram was constructed. As shown in Figure 2, 605 metabolites were identified in the four samples, among which 674, 671, 633, and 667 metabolites were identified in RAWJ, RIPEJ, HHLSJ, and MJ, respectively. Compared with the MJ group, 19, 21, and 14 unique metabolites were identified in the RAWJ, RIPEJ, and HHLSJ groups, respectively. Compared with the other three groups, the unique metabolites in RIPEJ were quercetagetin, apigenin-7-O-glucoside, and apigenin-4′-O-glucoside, and the unique metabolite in MJ was 2′-deoxyuridine 5′-monophosphate. The results of this study are consistent with those reported previously, further supporting that the addition of tea is beneficial to enrich the quality components of liquor [25].

A total of 109 other compounds were detected, which could be divided into eight subclasses, including alcohol compounds (0.72%), aldehyde compounds (1.45%), chromone (0.14%), ketone compounds (0.87%), lactones (0.72%), saccharides (6.08%), vitamins (0.43%), and others (5.35%). Among them, MJ had the highest number of species (109), followed by RAWJ (108), RIPEJ (108), and HHLSJ (102).

A total of 103 flavonoids were detected, which could be divided into seven subclasses, including chalcones (0.14%), flavanols (1.88%), flavanones (1.45%), flavones (4.2%), flavonols (5.5%), isoflavones (0.72%), and other flavonoids (1.01%). Among them, RIPEJ had the highest number of species (101), followed by RAWJ (99), MJ (94), and HHLSJ (89). Flavonoid compounds are bioactive components that are widely present in plants and fruits. The RAWJ and RIPEJ samples had more types of flavonoids compared to MJ. This is attributed to the fact that tea is rich in polyphenols [26]; therefore, the flavonoid types in tea liquor are higher than those in rice-flavor liquor.

A total of 90 lipid compounds was detected in the four samples, which could be divided into six subclasses: free fatty acids (9.12%), glycerol ester (0.29%), lysophosphatidylcholines (1.16%), lysophosphatidylethanolamines (0.58%), phosphatidylcholines (0.14%), and sphingolipids (1.74%). Among them, RIPEJ and RAWJ had the highest number of lipid species (88), followed by MJ (86) and HHLSJ (85).

A total of 77 alkaloids were detected, which could be divided into the following eight subclasses: alkaloids (7.53%), isoquinoline alkaloids (0.14%), phenolamine (0.58%), piperidine alkaloids (0.29%), plumerane (1.01%), pyridine alkaloids (0.43%), pyrrole alkaloids (0.72%), quinoline alkaloids (0.29%), and tropane alkaloids (0.14%). Among them, RAWJ had the highest number of species (76), followed by MJ (75), RIPEJ (72), and HHLSJ (71). Alkaloids are a class of natural nitrogen-containing alkaline organic compounds. Some alkaloids have a bitter taste [27], which will make the flavor of the liquor more rich and complex.

A total of 74 phenolic acids were detected. RAWJ had the largest number of species in this class (72), followed by RIPEJ (68), MJ (67), and HHLSJ (61). Phenolic acid is a plant metabolic product [28] and tea is known to be rich in phenolic acid compounds, such as gallic acid and salicylic acid. These compounds have well-established health functions such as anti-oxidation and free radical-scavenging activity [29]. As the main bioactive compounds in the brewing process, the primary and secondary materials under the action of various enzyme systems and microorganisms will also generate some phenolic acids and phenolic acid esters; these biologically active substances enter the liquor body through the unique distillation process, resulting in an elevated concentration in the final liquor products.

A total of 70 organic acids were detected, with the highest numbers found in MJ and RIPEJ (70), followed by RAWJ (68) and HHLSJ (65). Tea is rich in organic acids, including monocarboxylic acid and polycarboxylic acid [30]. Organic acids are precursors of ester synthesis, which are of great significance in regulating the wine body’s taste and also display health functions; in particular, esters can effectively reduce the bitterness of the liquor while increasing the sweetness. According to their chain length, organic acids can be divided into short-chain and long-chain acids. Short-chain acids have been associated with beneficial effects in treating alcoholism [31], whereas long-chain acids have been shown to reduce the risk of cardiovascular diseases while other acid compounds can protect biological systems from the influence of hydroxyl and peroxygen free radicals [32].

A total of 68 amino acids and their derivatives were detected with the highest numbers found in RIPEJ and HHLSJ (68), followed by MJ (67) and RAWJ (66). Amino acids and their derivatives are mainly generated from the fermentation process of grains, which contribute to the “sweet” and “fresh” tastes of liquor, along with the ability to moderate the sour and spicy taste of liquor [33]. Tea has a low content of free amino acids and rice flavor liquor has a high protein and amino acid content [34]. Given that the number of amino acids and their derivatives was highly similar among the four samples, the advantages of tea liquor with respect to the amino acid content are unclear.

A total of 51 kinds of terpenoids were detected, which were the most abundant in RAWJ, RIPEJ, and MJ (51), followed by HHLSJ (49). Terpenoids are the general term for all isoprene polymers and their derivatives, which can be divided into monoterpenoids, sesquiterpenoids, diterpenoids, triterpenoids, tetraterpenoids, or carotenes, which are characterized by a series of antibacterial and antioxidant properties [35]. In recent years, the research on terpenoids in liquor has become more extensive, and some studies have shown that terpenoids have important contributions to the aroma of liquor [36].

Compared to the other metabolites identified, the proportions of lignans and coumarins, nucleotides and their derivatives, quinones, and tannins in the four samples of liquor were lower, with only 22, 19, 6, and 2 of these metabolite types identified, respectively.

In summary, our results suggest that the mixed fermentation of tea and grain will not change the key flavor substances in liquor in general, and there is no obvious quantitative difference in substance types. Therefore, the appropriate use of tea and grain fermentation may impart unique flavor and aroma components to liquor.

### 3.2. Screening of Differential Metabolites

#### 3.2.1. Data Quality Assessment

**Figure 3:**
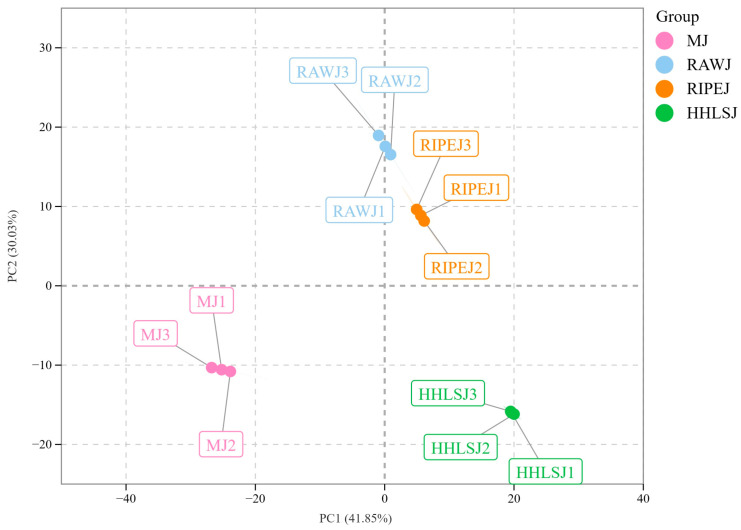
Principal component analysis of metabolites identified in four kinds of liquor samples.

To further understand the overall metabolic differences among the four samples and the differences among samples in the same group, PCA was conducted on all metabolites in the four samples. As shown in Figure 3, RAWJ, RIPEJ, HHLSJ, and MJ were significantly separated along PC1 (41.85%) and PC2 (30.03%). The RAWJ and RIPEJ samples were close to each other and the total contribution rate of cumulative variance in PC1 and PC2 was 71.88%. The results showed significant differences in metabolites between different tea liquors and rice-flavor liquor.

**Figure 4:**
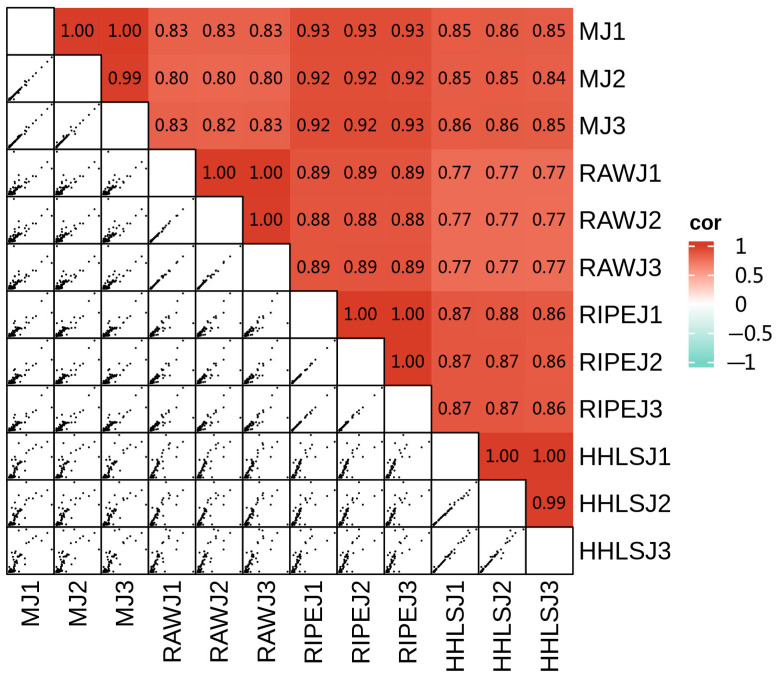
Correlation analysis between duplicated samples of the four kinds of liquor.

Correlation analysis was used as an evaluation index of bioreplicated correlation where different colors represent different correlation coefficients; where red indicates a stronger positive correlation (cor value closer to 1), white indicates a weaker correlation, and blue indicates a stronger negative correlation (cor value closer to 1). As shown in Figure 4, all samples had cor values close to 1, indicating credible biological duplication in the same group of samples.

#### 3.2.2. OPLS-DA Analysis

To further explore the differences in metabolite types of the four samples, the OPLS-DA method was used, which is a supervised statistical method of discriminant analysis. The key feature of OPLS-DA is that it can remove data changes unrelated to a categorical variable Y in an independent variable X so that the classification information is mainly concentrated along on one principal component [37]. R2X (cum) and R2Y (cum) are two parameters calculated by cross-validation procedures to evaluate the goodness of fit, while Q2 (cum) is a parameter used to describe the validity of the model [38]. In this study, Q2 = 0.997, R2X = 0.855, and R2Y = 1 in RAWJ and MJ; Q2 = 0.997, R2X = 0.856, R2Y = 1 in RIPEJ and MJ; and Q2 = 0.997, R2X = 0.896, and R2Y = 0.999 in HHLSJ and MJ, indicating that MJ is separated from the RAWJ, RIPEJ, and HHLSJ samples.

**Figure 5:**
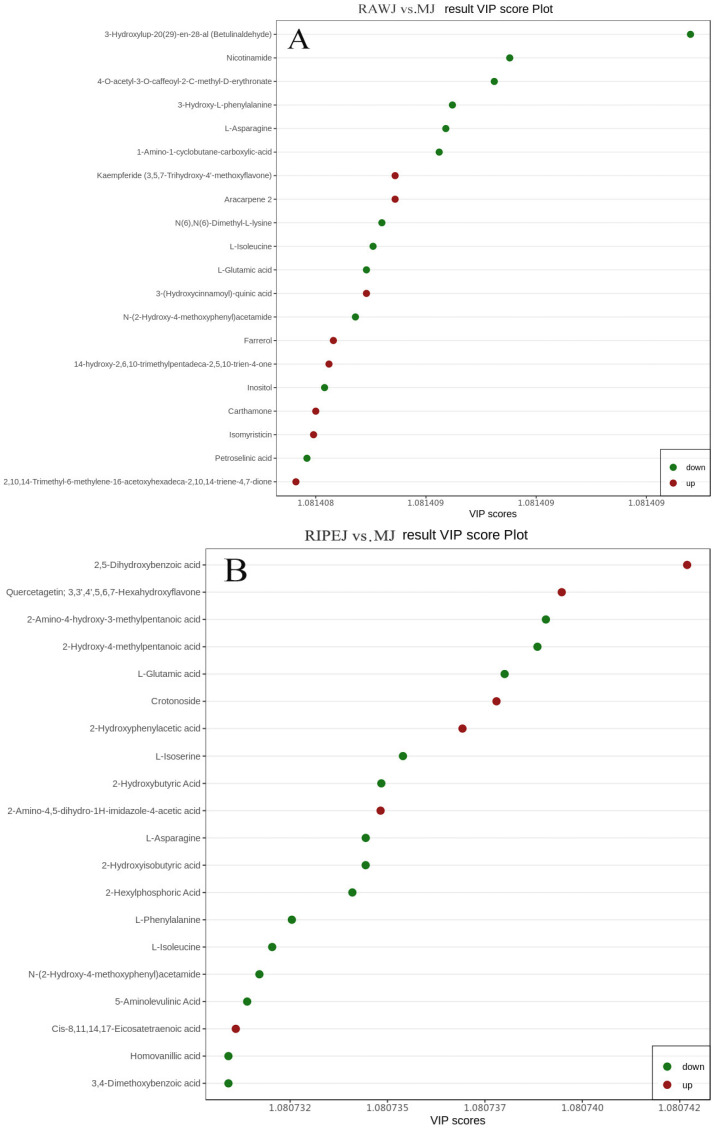

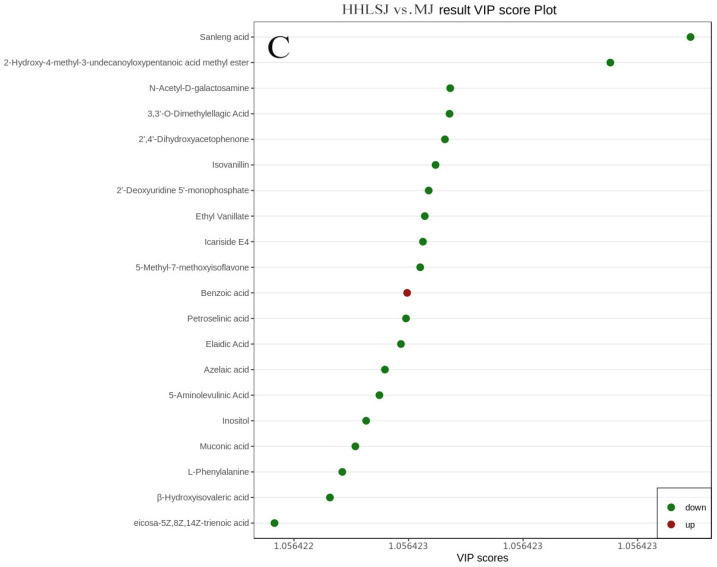
VIP values of metabolites compared between RAWJ and MJ (**A**), RIPEJ and MJ (**B**), and HHLSJ and MJ (**C**).

The top 20 metabolites with high VIP values in each group are shown in Figure 5. For example, 3-hydroxylup-20(29)-en-28-al, nicotinamide, 4-O-acetyl-3-O-caffeoyl-2-C-methyl-D-erythronate six amino acids and derivatives, six other compounds, four flavonoids, and one terpenoid, lipid, phenolic acid, and alkaloid were each identified in the RAWJ and MJ groups; 2,5-dihydroxybenzoic acid, quercetagetin, 2-amino-4-hydroxy-3-methylpentanoic acid, seven amino acids and derivatives, four organic acids, three phenolic acids, three alkaloids, and one flavonoid, other compounds, and lipid were each identified in the RIPEJ and MJ groups; and sanleng acid, 2-hydroxy-4-methyl-3-undecanoyloxypentanoic acid methyl ester, N-acetyl-D-galactosamine, five lipids, four other compounds, three organic acids, two phenolic acids, and one tannin, nucleotide, and derivative, lignan and coumarin, flavonoid, alkaloid, and amino acid and derivatives were each identified in the HHLSJ and MJ groups.

**Figure 6:**
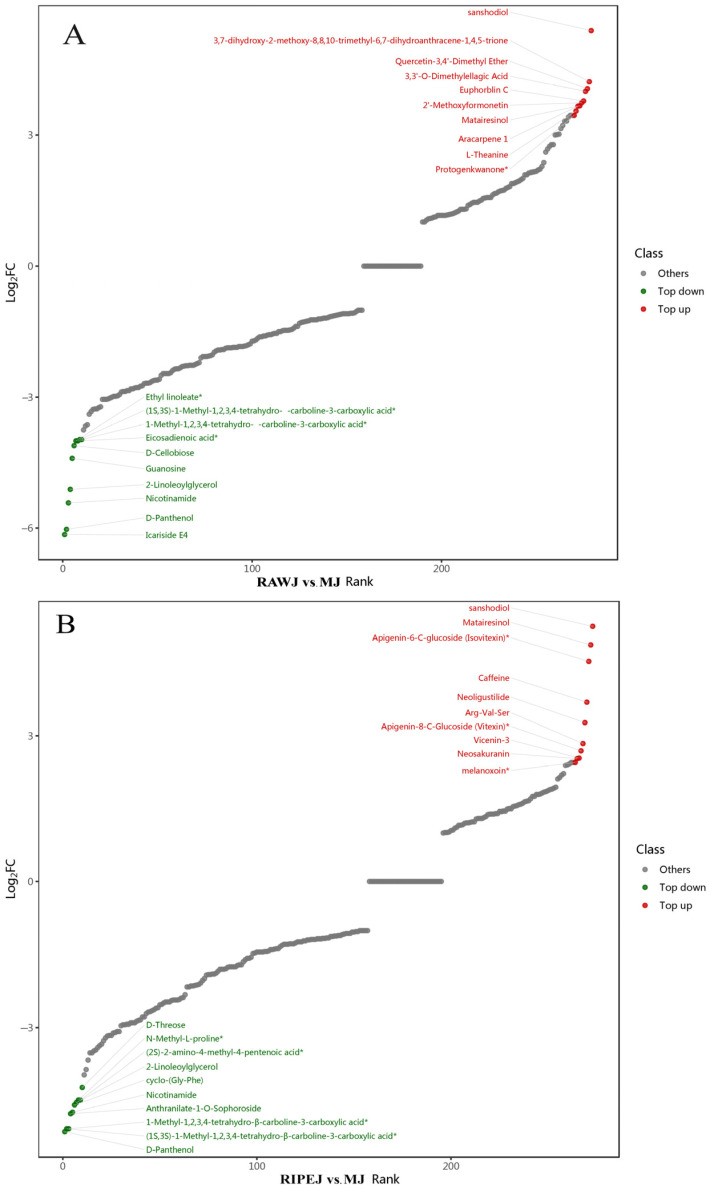

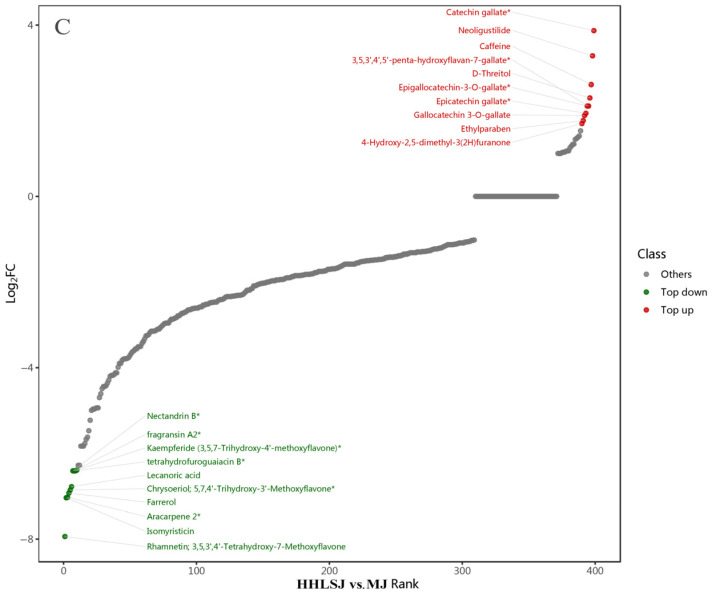
Dynamic distribution of metabolite content differences between RAWJ and MJ (**A**), RIPEJ and MJ (**B**), and HHLSJ and MJ (**C**). Note: The asterisk (*) in the diagram denotes the presence of isomers for the substance.

The FC in the metabolite contents of tea liquor and rice-flavor liquor was calculated to visually demonstrate their overall differential metabolite compositions. The metabolites were then arranged according to the FC values and a dynamic distribution diagram of metabolite content difference was generated (Figure 6). A total of 272, 246, and 125 metabolites with FC ≥ 1 or ≤0.5 were identified in RAWJ, RIPEJ, and HHLSJ samples compared with MJ, respectively. Figure 6 displays the top 10 up-regulated and down-regulated differential metabolites. For instance, sanshodiol showed the highest degree of up-regulation in RAWJ compared to MJ, while icariside E4 exhibited the lowest level of down-regulation. Similarly, sanshodiol had the highest up-regulation, while D-pantothenol had the lowest down-regulation in RIPEJ compared to MJ. In HHLSJ, epicatechin gallate was up-regulated with the highest FC, while rhamnetin was down-regulated with the lowest FC value compared to the corresponding levels in MJ.

**Figure 7:**
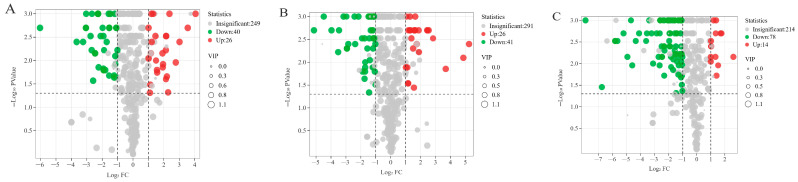
Volcano plots of differential nonvolatile metabolites for the comparisons of RAWJ vs. MJ (**A**), RIPEJ vs. MJ (**B**), and HHLSJ vs. MJ (**C**).

**Figure 8:**
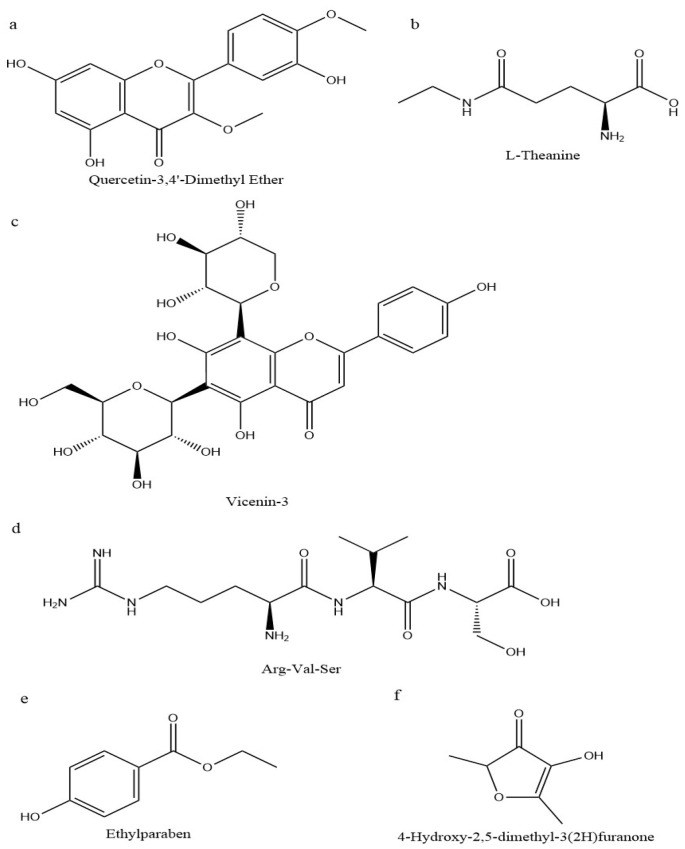
Simplified chemical structures of key differentiated substances in the three groups based on screening according to the fold change value, variable importance in projection (VIP) value, and *p*-value. (**a**,**b**) RAWJ vs. MJ; (**c**,**d**) RIPEJ vs. MJ; (**e**,**f**) HHLSJ vs. MJ.

To further comprehend the disparities in metabolites among RAWJ, RIPEJ, HHLSJ, and MJ, the FC values and VIP scores were used to identify significant differential metabolites among a total of 691 identified metabolites. According to the screening criteria (VIP ≥ 1; FC ≥ 2 or ≤0.5; *p* ≤ 0.05) [37], 655 significantly distinct metabolites were identified (Table S1). The volcano plots for each group are depicted in Figure 7, with red and green dots denoting up-regulated and down-regulated differentiated metabolites, respectively, while gray dots represent non-differentiated metabolites among groups. In the RAWJ and MJ groups (315 species), 26 species were up-regulated and 40 species were down-regulated (Figure 7A). In the comparison of the RIPEJ and MJ groups (358 species), 26 were up-regulated and 41 were down-regulated (Figure 7B). In the comparison of the HHLSJ and MJ groups (306 species), 14 were up-regulated and 78 were down-regulated(Figure 7C). Statistically significant differences were found in the types of substances in each group, totaling 60 flavonoids, 34 phenolic acids, 34 others, 22 lipids, 21 alkaloids, 10 amino acids and their derivatives, 10 nucleotides and their derivatives, 9 terpenoids, 5 quinones, 4 lignans, and coumarins compounds. Among these, flavonoids showed the most important difference between tea wine and rice wine. The chemical structural formulas of the metabolites with significant differences in content in each group are presented in Figure 8.

Finally, we evaluated the differences in the relative contents of non-volatile metabolites in different groups according to the unit variance scale. According to the K-means clustering method, 529 different metabolites were divided into seven subclasses (Table S2). There were 82 metabolites in RAWJ with higher contents than those found in MJ, RIPEJ, and HHLSJ. In this subgroup, the most common metabolites were flavonoids (29 species), including kaempferide and quercetin-3-O-glucoside. In subclass 2, the contents of 53 metabolites in MJ and RAWJ were higher than those in RIPEJ and HHLSJ, of which 12 were lipids, hydroperoxylinoleic acid, 9-hydroxy-12-oxo-15(Z)-octadecenoic acid, and others. In subclass 3 and subclass 6, the contents of 140 and 88 metabolites in MJ were higher than those in RAWJ, RPIEJ, and HHLSJ, including L-asparagine, DL-threonine, L-glutamine, and 46 other amino acids and derivatives. In subclass 4, the contents of 28 metabolites in HHLSJ were higher than those in MJ, RAWJ, and RIPEJ, with the majority represented by flavonoids (eight kinds), including catechin (+C), epicatechin gallate, and epigallocatechin-3-O-gallate. In subclass 5, 98 metabolites were found at higher abundance in RIPEJ than in MJ, RAWJ, and HHLSJ, which were also dominated by flavonoids (34 species), such as kaempferol-3-O-robinobioside(biorobin), kaempferol-3-O-glucoside-7-O-rhamnoside, and 4′,6-dihydroxyflavone. In subclass 7, a total of 40 metabolites were found at lower levels in RAWJ than in MJ, RAWJ, and HHLSJ, with alkaloids (eight species) being the most abundant, including betaine and acetaminophen. Among these, acetaminophen is mainly derived from chemical synthesis and is not found in natural products. Its detection in the sample may be due to contamination of the experimental materials or labeling errors.

## 4. Conclusions

In this study, based on the broadly targeted metabolomics method of UPLC-MS/MS, we identified differences in the metabolite composition between three types of tea liquor and rice-flavor liquor. A total of 691 metabolites were identified in the four liquor samples (RAWJ, RIPEJ, HHLSJ, and MJ), including 109 “other” metabolite types, 103 flavonoids, 90 lipids, 77 alkaloids, 74 phenolic acids, 70 organic acids, 68 amino acids and derivatives, 51 terpenoids, 22 lignans and coumarins, 19 nucleotides and derivatives, 6 quinones, and 2 tannins. The tea liquors RAWJ, RIPEJ, and HHLSJ had an additional 19, 21, and 14 unique metabolites compared with those found in the MJ sample, respectively. Flavonoids are the most important differential metabolites between tea liquor and rice-flavor liquor. This study systematically revealed the composition of non-volatile substances of tea liquor, which can provide a certain theoretical basis for further in-depth research and development of tea liquor.

## Data Availability

The original contributions presented in the study are included in the article and Supplementary Materials, further inquiries can be directed to the corresponding author.

## Supplementary Materials

The following supporting information can be downloaded at https://www.mdpi.com/article/10.3390/foods13172800/s1: Figure S1 (a): MRM detection of multimodal maps positive Ion Mode; (b): MRM detection of multimodal maps negative ion mode; Figure S2 (a): QC_MS_tic_overlap-N; (b): QC_MS_tic_overlap-P; (c): QC_MS_TIC-N; (d): QC_MS_TIC-P; (e): Catechin second-order spectrum; (f): L-Theanine second-order spectrum; (g): Epicatechin gallate second-order spectrum; (h): Gallocatechin 3-O-gallate second-order spectrum; (i): Vicenin-3 second-order spectrum; (j): Epigallocatechin-3-O-gallate second-order spectrum; (k): Epicatechin second-order spectrum; Table S1: Metabolite Profiles Identified by FC, VIP, and P-Value Screening; Table S2: Metabolite Clustering Results from K-means Analysis.
