# Supplementary figures and images for: Analysis of Metabolite Differences in Different Tea Liquors Based on Broadly Targeted Metabolomics
Source: Foods. 2024 Sep 3;13(17):2800. doi: 10.3390/foods13172800 (PMC11394687; doi:10.3390/foods13172800)

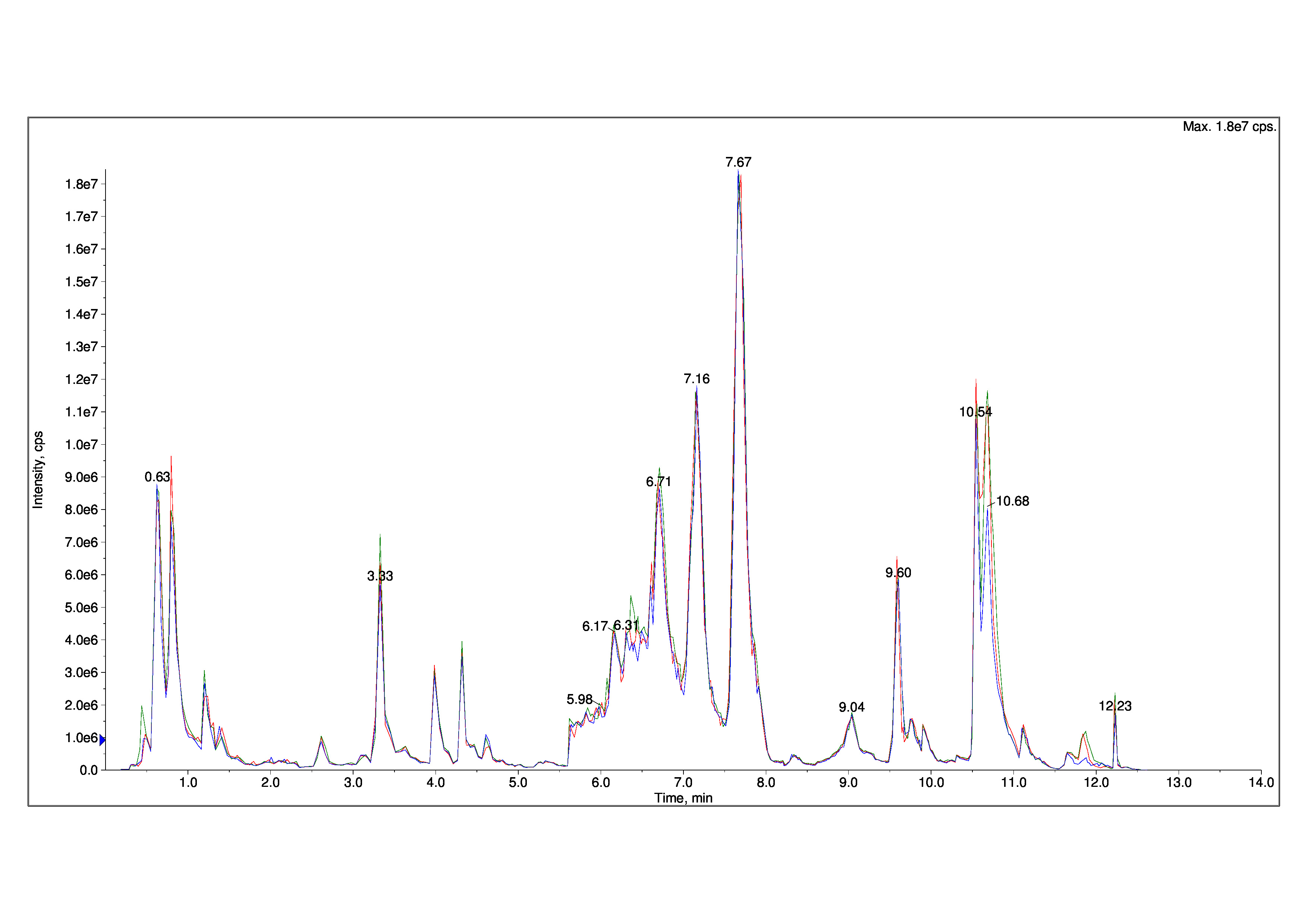

Supplement: Supplementary file 1 [file foods-13-02800-s001.zip › Splment_figure 2a QC_MS_tic_overlap-N.jpg]

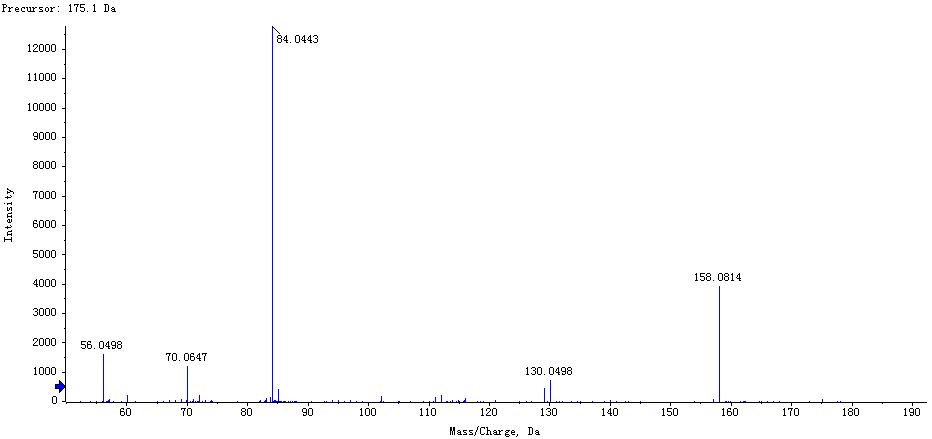

Supplement: Supplementary file 1 [file foods-13-02800-s001.zip › Suppelmentary figure 2f L-Theanine.jpg]

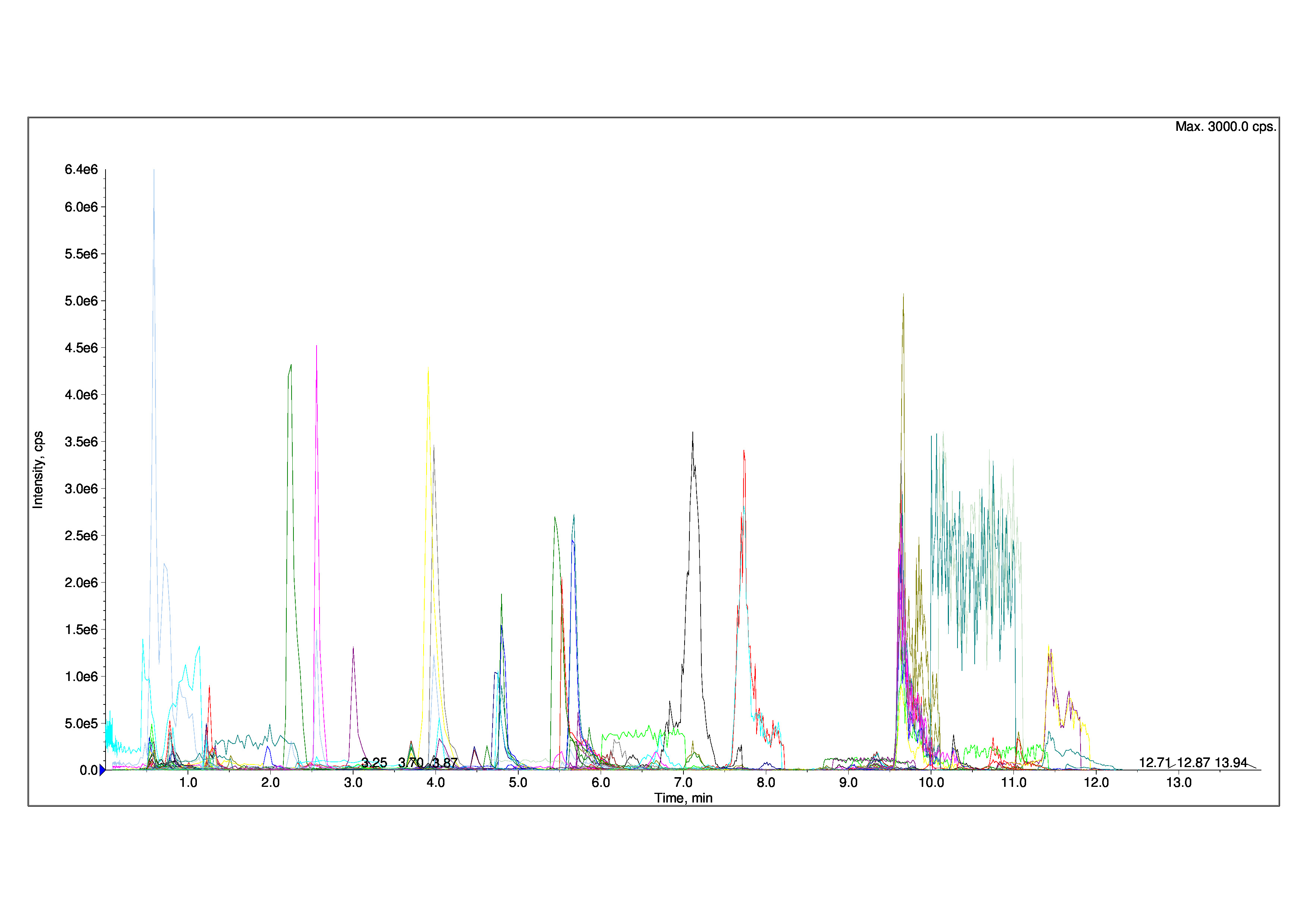

Supplement: Supplementary file 1 [file foods-13-02800-s001.zip › Supplementary figure 1a-MRM detection of multimodal maps positive Ion Mode.jpg]

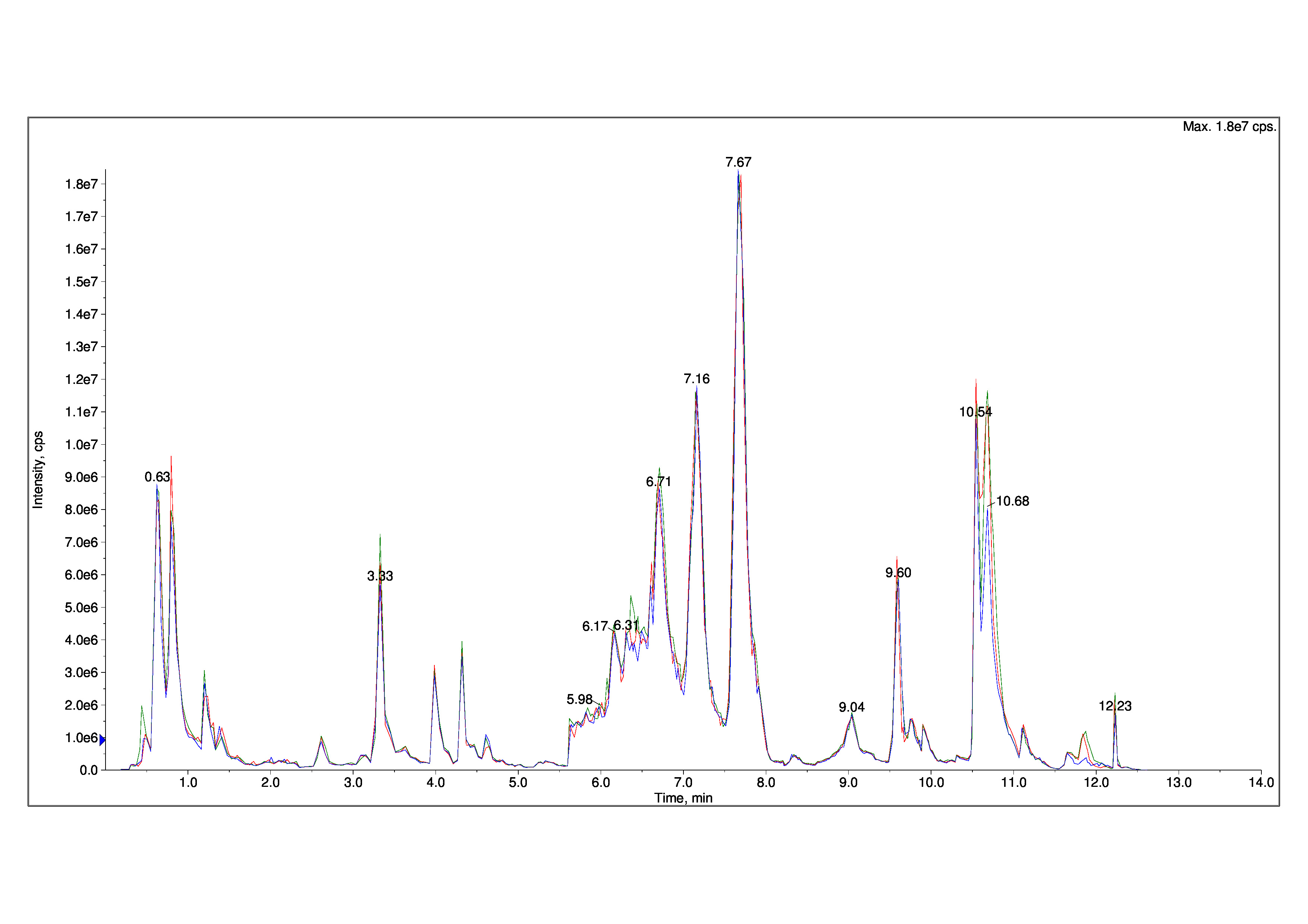

Supplement: Supplementary file 1 [file foods-13-02800-s001.zip › Supplementary figure 1b- MRM detection of multimodal maps negative ion mode.jpg]

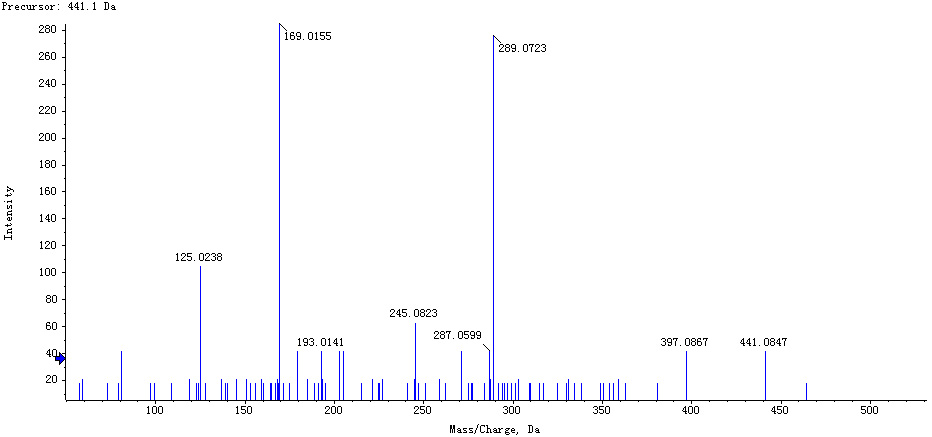

Supplement: Supplementary file 1 [file foods-13-02800-s001.zip › Supplementary figure 2 g Epicatechin gallate.jpg]

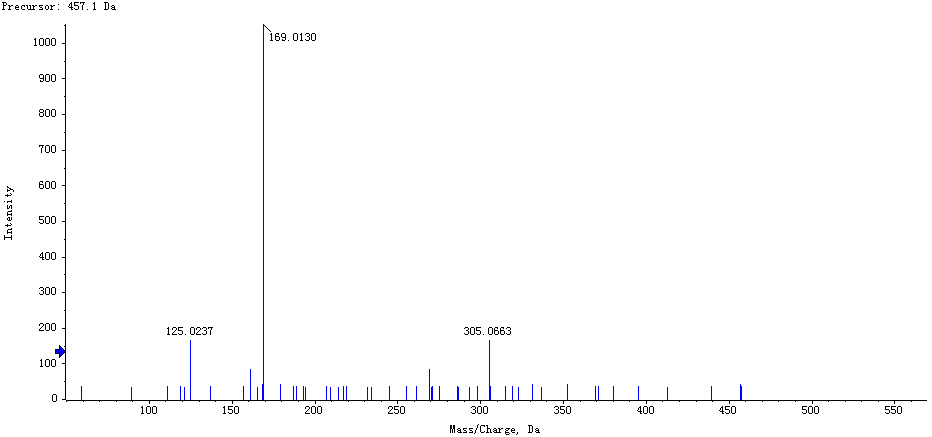

Supplement: Supplementary file 1 [file foods-13-02800-s001.zip › Supplementary figure 2 h Gallocatechin 3-O-gallate.jpg]

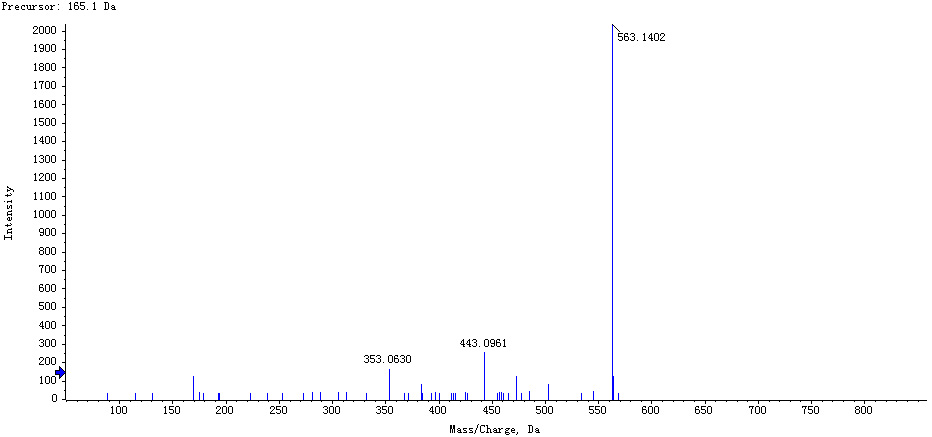

Supplement: Supplementary file 1 [file foods-13-02800-s001.zip › Supplementary figure 2 i Vicenin-3.jpg]

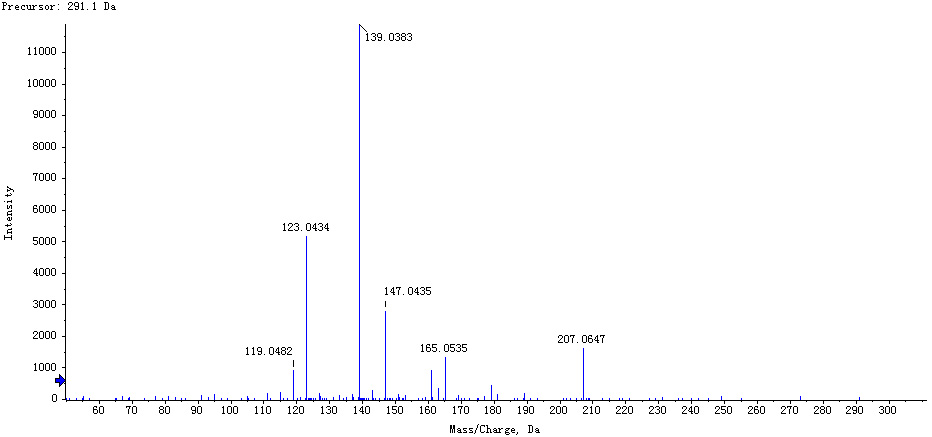

Supplement: Supplementary file 1 [file foods-13-02800-s001.zip › Supplementary figure 2 k Epicatechin.jpg]

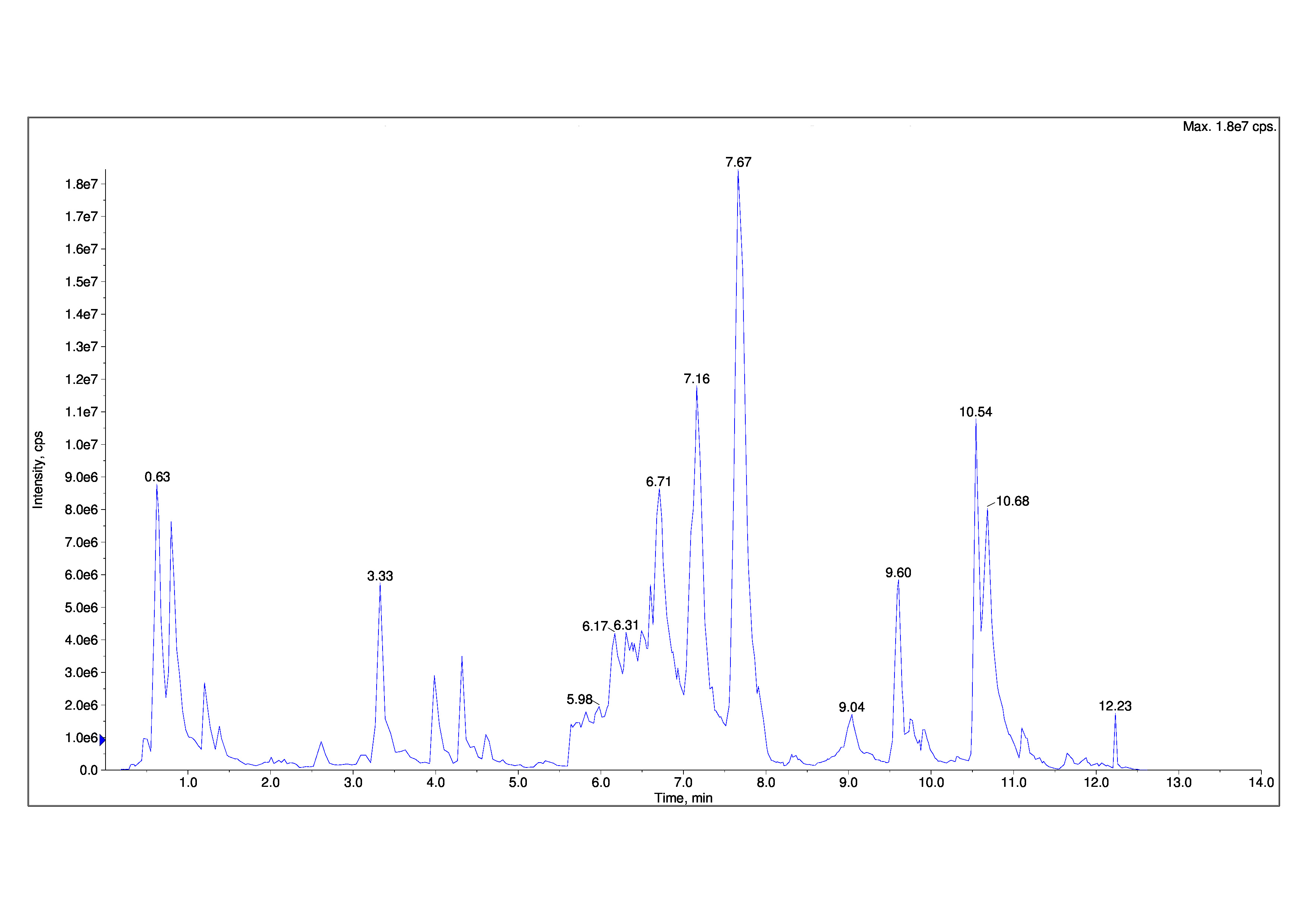

Supplement: Supplementary file 1 [file foods-13-02800-s001.zip › Supplementary figure 2c QC_MS_TIC-N.jpg]

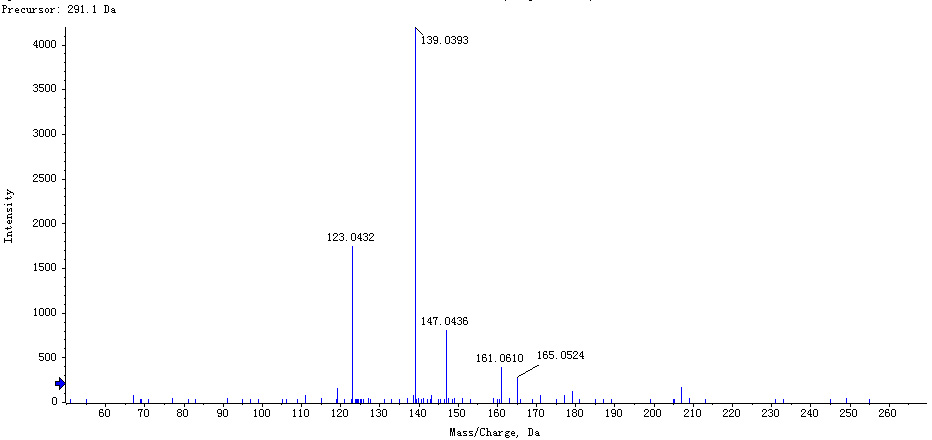

Supplement: Supplementary file 1 [file foods-13-02800-s001.zip › Supplementary figure 2e Catechin.jpg]

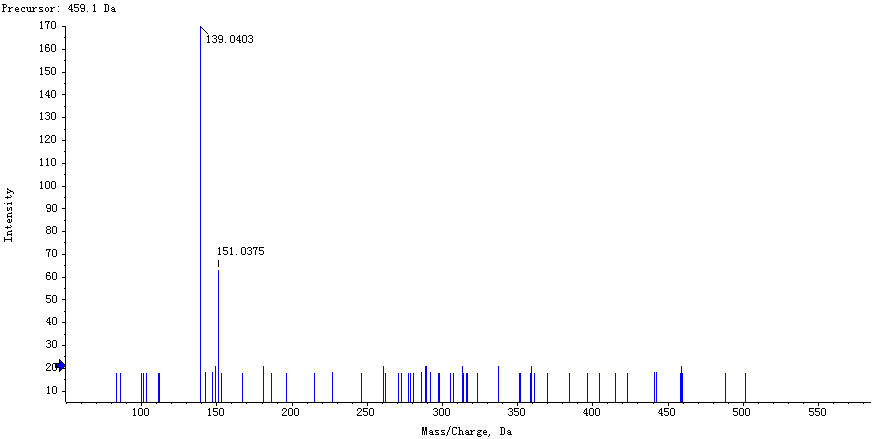

Supplement: Supplementary file 1 [file foods-13-02800-s001.zip › Supplementary figure 2j Epigallocatechin-3-O-gallate.jpg]

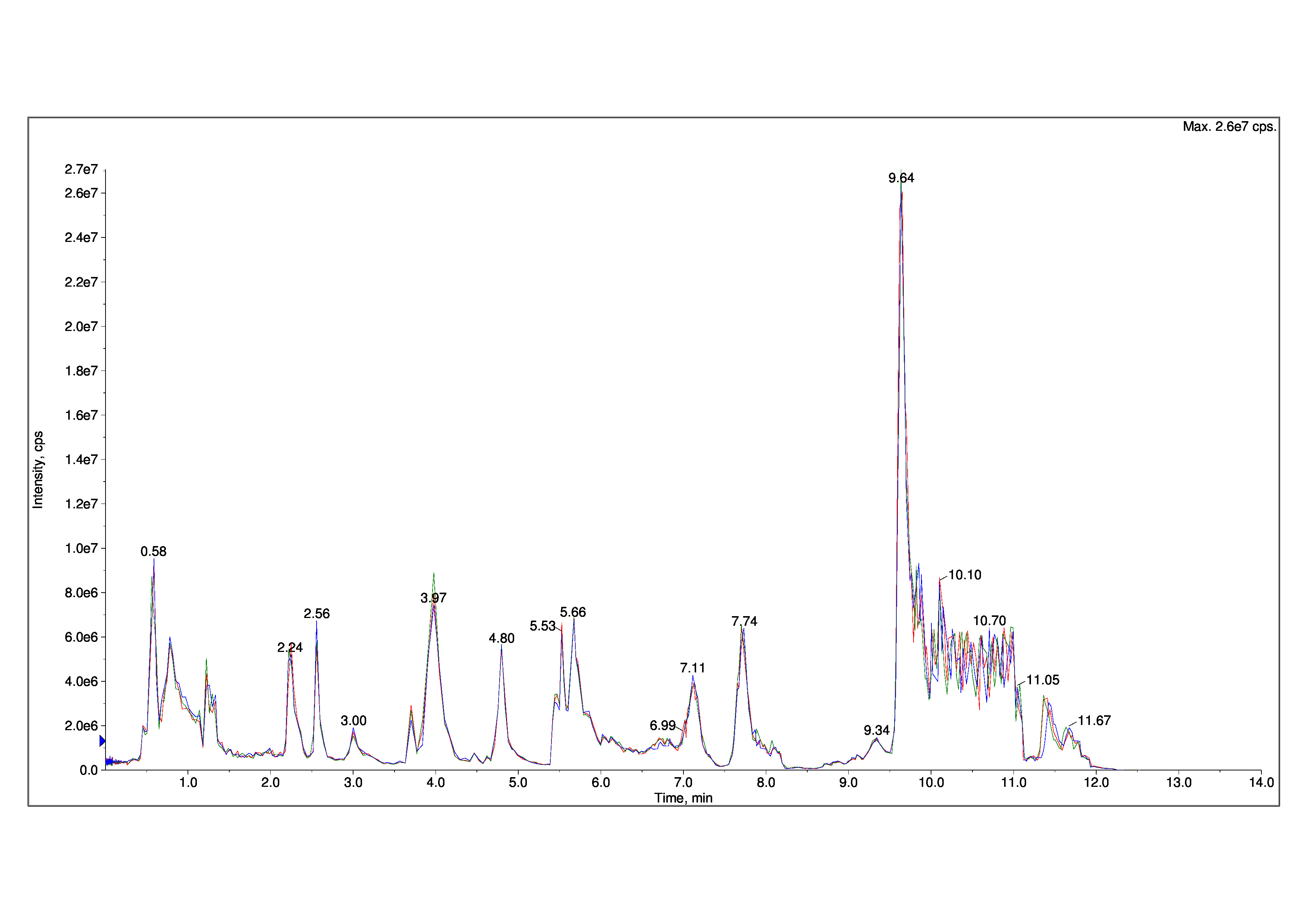

Supplement: Supplementary file 1 [file foods-13-02800-s001.zip › Supplementary figure_2b QC_MS_tic_overlap-P.jpg]

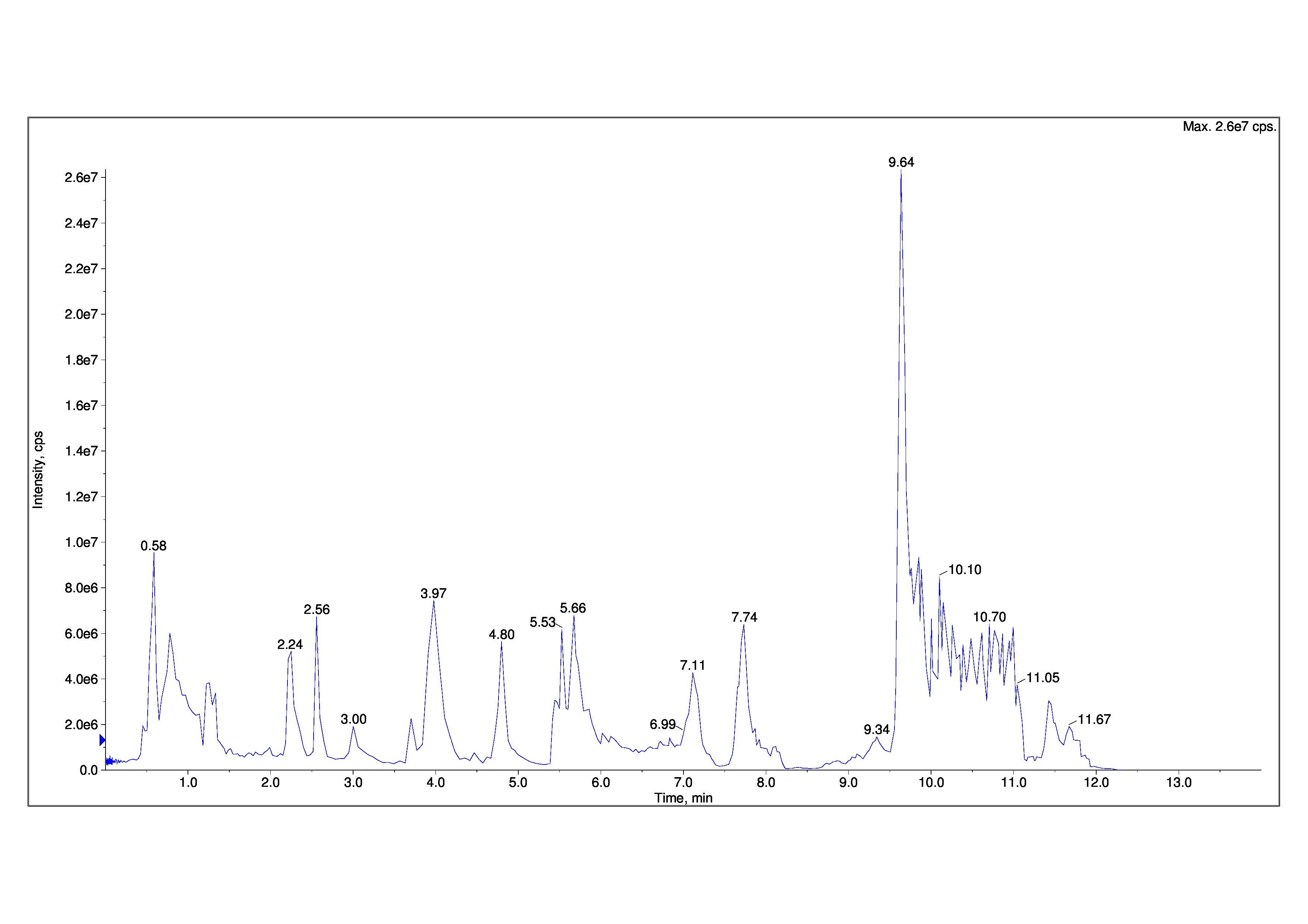

Supplement: Supplementary file 1 [file foods-13-02800-s001.zip › Supplementary figure_2d QC_MS_TIC-P.jpg]
